# Psychological determinants of diabetes self-management and quality of life: A cross-sectional study in Greece

**DOI:** 10.1192/j.eurpsy.2026.11042

**Published:** 2026-07-31

**Authors:** P. Gkaragkani, A. Papadopoulou, P.-D. Stavrou, E. Vousoura, V. Efstathiou

**Affiliations:** 1 MSc in Health Promotion and Education, School of Medicine, National and Kapodistrian University of Athens; 2 Second Department of Psychiatry, “Attikon” University General Hospital, National and Kapodistrian University of Athens; 3 Department of Psychology, National and Kapodistrian University of Athens, Athens, Greece

## Abstract

**Introduction:**

Diabetes self-management requires complex lifestyle adjustments and sustained adherence to treatment. Psychological determinants, such as diabetes-related distress, self-efficacy, perceived health, and the doctor–patient relationship, have been identified as key influences on self-care behaviors and quality of life. Despite growing evidence, findings regarding their impact on self-management and quality of life remain inconsistent.

**Objectives:**

This study aimed to examine the extent to which psychological factors (diabetes distress, anxiety and depression, attitudes towards the doctor-patient relationship, knowledge about diabetes, self-efficacy, health perceptions, and social support) are related to self-management of diabetes mellitus patients, and their influence on self-care activities and quality of life.

**Methods:**

A Greek sample of 112 adults with diabetes completed questionnaires assessing self-care (Diabetes Self-Care Activities Questionnaire), quality of life (Diabetes Quality of Life Brief Clinical Inventory), diabetes-related distress (Diabetes Distress Scale), anxiety and depression (Patient Health Questionnaire), knowledge (Diabetes Knowledge Questionnaire), self-efficacy (Diabetes Management Self-Efficacy Scale), illness perceptions (Brief Illness Perception Questionnaire), doctor–patient relationship (Patient–Practitioner Orientation Scale), and social support (Multidimensional Scale of Perceived Social Support). Analyses included parametric and non-parametric tests, correlation analyses, and mediation analyses.

**Results:**

Self-care did not mediate relationships between psychological factors and quality of life. Participants showed basic knowledge (M ± SD=16.2 ± 3.5, min/max=6–22) and moderate distress (2.3 ± 1.3, min/max=1–5.1). Self-care was low for exercise and complication prevention but sufficient for glucose monitoring (84.6 ± 11.8, min/max=58–108). Emotional distress was frequent; quality of life was moderate (51.8 ± 9.6, min/max=35–63). A positive doctor-patient relationship was reported (62.6 ± 7.2, min/max=42–80). Self-efficacy was higher in everyday situations but lower under stress. Family was the main source of social support (67.3 ± 14.7, min/max=12–84). Self-care did not differ by education (p=0.752) or gender (p=0.868). Improved foot care and glucose testing (r=0.405, p<0.001) were linked to longer disease duration. Participation in educational programs was associated with better diet, foot care, and exercise. Quality of life correlated negatively with distress (r=–0.336, p=0.001). No psychological factor showed a significant direct or indirect effect on quality of life through self-care.

**Conclusions:**

Self-care improved with longer disease duration and educational programs, while quality of life was inversely related to distress. These findings underscore the need for educational interventions and psychological support to strengthen diabetes self-management.

**Disclosure of Interest:**

None Declared

